# Echo Model Analysis and Frequency-Domain Imaging Algorithm for Geosynchronous Spaceborne–Airborne FMCW Bistatic SAR with High-Maneuvering Receiver

**DOI:** 10.3390/jimaging12070310

**Published:** 2026-07-08

**Authors:** Xinyu Liu, Li Ding, Chenlei Lu, Wenlong Yang, Ping Li

**Affiliations:** 1 School of Health Science and Engineering, University of Shanghai for Science and Technology, Shanghai 200093, China; 243352492@st.usst.edu.cn (X.L.); sunnylding@usst.edu.cn (L.D.); 2 School of Optical-Electrical and Computer Engineering, University of Shanghai for Science and Technology, Shanghai 200093, China; 242200366@st.usst.edu.cn (C.L.); 243350728@st.usst.edu.cn (W.Y.)

**Keywords:** geosynchronous spaceborne–airborne bistatic synthetic aperture radar (GEO SA BiSAR), frequency-modulated continuous-wave (FMCW), high-maneuvering platform, acceleration model, frequency-domain imaging algorithm

## Abstract

Geosynchronous spaceborne–airborne frequency-modulated continuous-wave bistatic synthetic aperture radar (GEO SA FMCW BiSAR) offers cost-effective and persistent target monitoring. However, both the maneuvers of the receiver during the signal propagation delay and the continuous movements of the radar platforms within the sweep complicate the received echo signal. These factors invalidate the “stop-and-go” assumption, which presumes constant-velocity motion. This paper proposes an echo model that simultaneously considers intra-pulse motion and accelerated motion of the high-maneuvering receiver. The introduction of receiver acceleration leads to nonlinear range terms in the bistatic range history, which will degrade the focusing performance if not properly compensated. Since the acceleration term is a small second-order quantity relative to the time delay, it is approximated by segmenting the aperture and applying the “stop-and-go” assumption within each sub-aperture. After dechirp, the two-dimensional (2-D) spectrum for imaging is derived by applying the principle of stationary phase and determining the azimuth stationary phase point via series reversion. Finally, imaging is achieved by azimuth compression, range cell migration correction, and secondary range compression. Simulation results demonstrate that the proposed algorithm achieves well-focused images while maintaining computational efficiency.

## 1. Introduction

Geosynchronous spaceborne–airborne bistatic synthetic aperture radar (GEO SA BiSAR) leverages a high-orbit satellite for continuous, wide-area illumination [1,2,3,4,5,6], while employing an airborne receiver to enable flexible bistatic configurations for various applications [7,8,9,10,11]. When integrated with frequency-modulated continuous wave (FMCW) technology, the system benefits from lower transmit power and simplified hardware requirements compared with pulse-mode synthetic aperture radar (SAR) [12,13]. Despite these advantages, the imaging performance of this complex configuration is fundamentally determined by the development of an accurate radar echo model [14].

Conventionally, radar echo modeling relies on the “stop-and-go” assumption, which significantly simplifies the theoretical analysis of SAR imaging [15,16]. However, applying this assumption to GEO SA FMCW BiSAR encounters two challenges. On the one hand, due to the approximately 36,500 km altitude of the geosynchronous Earth orbit (GEO) satellite, the transmitter-to-target propagation delay reaches the order of seconds [17]. Consequently, the high-maneuvering motion of the airborne receiver during the propagation delay introduces higher-order phase errors into the echo signal. This motion violates the “stop-and-go” assumption that radar platforms remain stationary between pulse transmission and reception [18]. To mitigate these effects, research has been dedicated to developing advanced echo models and imaging algorithms. Reference [19] proposed a generalized “non-stop-and-go” echo model for bistatic radar configurations with high-maneuvering platforms. By incorporating higher-order motion terms of the receiver (e.g., acceleration and jerk) into the range history equation, a high-precision analytical expression for the signal propagation delay is derived. This extends the applicability of the model from conventional constant-velocity platforms to highly maneuvering ones. Reference [20] presented an improved propagation delay model for geosynchronous Earth orbit–low Earth orbit (GEO-LEO) bistatic configurations. The model fully accounts for both the long propagation delay induced by the high-orbit transmitter and the high-maneuvering characteristics of the low Earth orbit (LEO) receiver. By deriving an analytical expression for the propagation delay equation and validating the accuracy of the approximate solution, An et al. [20] developed a third-order propagation delay model. This model satisfies the signal accuracy requirements of geosynchronous spaceborne bistatic synthetic aperture radar (GEO BiSAR) and lays the foundation for precise imaging of GEO BiSAR. Reference [21] further proposed a precise third-order range model for GEO SA BiSAR based on the “non-stop-and-go” model. The model separately analyzes the transmitter and receiver delays while accounting for the motion of both the target and the receiver. The theoretical analysis demonstrates the necessity of considering the “non-stop-and-go” effect to achieve high-precision imaging in GEO SA BiSAR configurations. However, the aforementioned studies on the “non-stop-and-go” model primarily focus on pulsed SAR systems. Due to the substantial difference in the waveform, the pulsed SAR echo model is inapplicable to FMCW SAR.

On the other hand, due to the characteristics of FMCW [22], the intra-pulse motion of the transmitter and receiver introduces the influence of the extra Doppler frequency shift and range migration [23,24], as well as the problem of severe range–azimuth coupling [25]. This conflicts with the “stop-and-go” assumption, which considers only the slant range variation between radar platforms and the target at discrete azimuthal instants. As for FMCW BiSAR systems, significant progress has been made in modeling and imaging. Reference [26] simplifies the bistatic FMCW SAR range model to a single monostatic-like model by the Fresnel approximation. Furthermore, an intermediate-frequency signal model is derived, which enables precise imaging under the constant-velocity assumption. To address the impact of continuous platform motion during the pulse in spaceborne bistatic configurations, reference [27] proposed iterative methods to determine the time delay and thereby constructed a “non-stop-and-go” echo model. Despite these advancements, the combination of GEO-scale delays and the maneuverability of the airborne receiver in FMCW configurations still requires further investigation. As for the focusing methods, time-domain algorithms offer excellent model adaptability and high imaging precision. However, their substantial computational burden undermines the lightweight and cost-effective advantages of FMCW SAR systems. Concurrently, they fail to meet the real-time processing requirements of spaceborne or airborne radars due to their low computational efficiency. Consequently, they are hardly applicable in practice to the GEO SA FMCW BiSAR system.

Table 1 summarizes previous studies on “non-stop-and-go” echo models and imaging algorithms, comparing their capabilities and limitations. The comparison indicates that existing echo models for high-maneuvering BiSAR mainly focus on pulsed waveforms. Meanwhile, existing FMCW BiSAR models generally assume constant-velocity motion or employ a computationally expensive time-domain algorithm. In particular, the joint consideration of GEO-scale propagation delay, intra-sweep continuous motion, and receiver acceleration within a unified frequency-domain imaging framework remains insufficiently investigated in the existing literature. Different from existing studies, which either neglect receiver acceleration or rely on time-domain processing, the proposed approach incorporates the dynamic characteristics of the highly maneuvering receiver within the GEO SA FMCW BiSAR system into a frequency-domain formulation.

The main novelty of this work lies in the development of a “non-stop-and-go” echo model and its corresponding two-dimensional (2-D) frequency-domain imaging algorithm for high-maneuvering GEO SA FMCW BiSAR systems. The main contributions of our work are as follows:

1. In the proposed echo model, to ensure applicability to FMCW radar, the continuous motion of both the transmitter and receiver during the whole pulse repetition period is considered, along with the linear motion and nonlinear maneuvers of the receiver throughout the propagation of the transmitted signal. Subsequently, the high-order terms of the slant range history of the receiver are approximated to derive a precise mathematical solution for the propagation delay.
2. Based on the proposed model, an improved frequency-domain imaging algorithm is developed. In this paper, the dechirp operation of the raw echo in the 2-D time domain enables the time–frequency transformation in the range direction, with residual video phase (RVP) compensation to avoid defocusing in the final image. After realizing the time–frequency transformation in the azimuth direction through the method of series reversion (MSR), phase compensation is performed to realize range cell migration correction (RCMC), secondary range compression (SRC), and the elimination of high-order range–frequency coupling; finally, the 2-D focus is realized.

A list of the acronyms used in this paper is presented in Table 2. The paper is structured as follows. Section 2 presents the echo model of the GEO SA FMCW BiSAR system; Section 3 presents the derivation of the proposed algorithm; Section 4 presents simulation results regarding both targets and practical implementation viability; and Section 5 concludes this paper.

## 2. GEO SA FMCW BiSAR Echo Model

### 2.1. The Geometric Model

**Figure 1:**
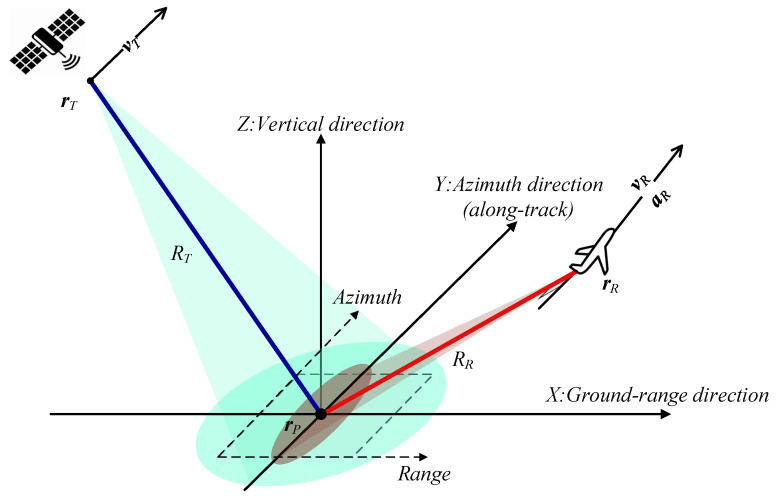
The geometric model of the GEO SA FMCW BiSAR system.

The geometric model of the GEO SA FMCW BiSAR system is shown in Figure 1. The transmitter is located on the high-orbit spaceborne platform, and the receiver is located on a high-maneuvering airborne platform. We establish a Cartesian coordinate system in which the Z-axis represents the vertical direction, the X-axis represents the ground-range direction, and the Y-axis represents the azimuth (along-track) direction, rather than the geographic azimuth angle, and is aligned with the direction of the flight path of the receiver. In this paper, “azimuth” denotes the slow-time dimension used in SAR. Here, $r_{T}=\left(x_{T},y_{T},z_{T}\right)$ represents the position vector of the transmitting radar; $v_{T}=\left(0,v_{T},0\right)$ represents the velocity vector of the transmitting radar; $r_{R}=\left(x_{R},y_{R},z_{R}\right)$ represents the position vector of the receiver; $v_{R}=\left(0,v_{R},0\right)$ represents the velocity vector of the receiver. In this paper, to illustrate the impact of maneuverability, we focus on a case with vertical acceleration $a_{R}=\left(0,0,a_{z}\right)$. This choice is justified by the following factors. First, vertical acceleration is used to represent realistic flight states, including ascent, descent, and vertical maneuvers. Second, isolating the vertical component aims to construct a benchmark scenario to evaluate the compensation capability of the proposed algorithm. It is worth noting that this simplification does not restrict the generality of our work. The same derivation applies to along-track or cross-track acceleration components because the acceleration vector enters the range history without directional sensitivity.

### 2.2. The Echo Model

**Figure 2:**
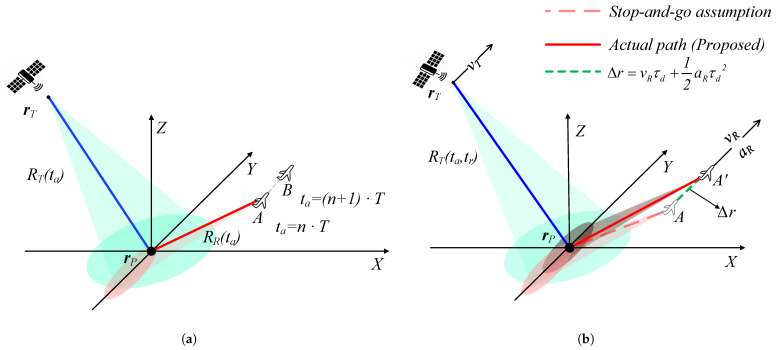
The echo model of GEO SA FMCW BiSAR, where *A* and *B* are the airborne receiver positions under the “stop-and-go” assumption. *n* and n+1 denote the pulse index. *T* denotes the pulse repetition interval. $A^{\prime }$ is the airborne receiver position under the actual path. Δr denotes the movement of the receiver during propagation delay. (**a**) The echo model under the “stop-and-go” assumption. (**b**) The actual echo model.

It is well known that the propagation delay is the foundation of the echo model. Under the “stop-and-go” assumption, as shown in Figure 2a, the radar platforms are considered stationary during the transmission and reception of each entire pulse. Additionally, platform positions are updated only at each slow-time instant. $R_{T}\left(t_{a}\right)$ and $R_{R}\left(t_{a}\right)$ denote the range histories of the transmitter and the receiver, respectively. The calculation equations are as follows:

(1) $$R_{T}\left(t_{a}\right)=\left|r_{T}+v_{T}t_{a}-r_{P}\right|$$

(2) $$R_{R}\left(t_{a}\right)=\left|r_{R}+v_{R}t_{a}-r_{P}\right|$$

Therefore, the propagation delay of the echo model under the “stop-and-go” assumption (called $\tau_{SAG}$) is expressed as

(3) $$\tau_{SAG}\left(t_{a}\right)=\frac{R_{T}\left(t_{a}\right)+R_{R}\left(t_{a}\right)}{c}$$

where $\tau_{SAG}$ denotes the propagation delay based on the “stop-and-go” assumption while *c* denotes the speed of light and $t_{a}$ denotes the slow-time variable. However, in the GEO SA FMCW BiSAR system with a slant range of approximately 37,000 km, the signal round-trip propagation delay is on the order of seconds. During this long duration, the displacement of the high-maneuvering receiver is significant; therefore, Equation (3) should consider the movement of the receiver when the signal is propagating.

Figure 2b shows the actual echo model (“non-stop-and-go”) for GEO SA FMCW BiSAR, accounting for the coupling effects of fast-time and slow-time on radar platform motion. The actual propagation delay (called $\tau_{d}$) is given as follows:

(4) $$\tau_{d}\left(t_{a},t_{r}\right)=\frac{R_{T}\left(t_{a},t_{r}\right)+R_{R}\left(t_{a},t_{r};\tau_{d}\left(t_{a},t_{r}\right)\right)}{c}$$

where $t_{r}$ denotes the fast-time variable. Furthermore, $R_{T}\left(t_{a},t_{r}\right)$ denotes the range history of the transmitter. $R_{R}\left(t_{a},t_{r};\tau_{d}\left(t_{a},t_{r}\right)\right)$ denotes the range history of the receiver, accounting for its motion during the actual propagation delay. The high-maneuvering motion of the receiver is modeled by introducing additional acceleration. The range histories are expressed by the following equations:

(5) $$R_{T}\left(t_{a},t_{r}\right)=\left|r_{T}+v_{T}\left(t_{a}+t_{r}\right)-r_{P}\right|$$

(6) $$R_{R}\left(t_{a},t_{r};\tau_{d}\left(t_{a},t_{r}\right)\right)=\left|r_{R}+v_{R}\left(t_{a}+t_{r}+\tau_{d}\left(t_{a},t_{r}\right)\right)+a_{R}{\left(t_{a}+t_{r}+\tau_{d}\left(t_{a},t_{r}\right)\right)}^{2}/2-r_{P}\right|$$

**Figure 3:**
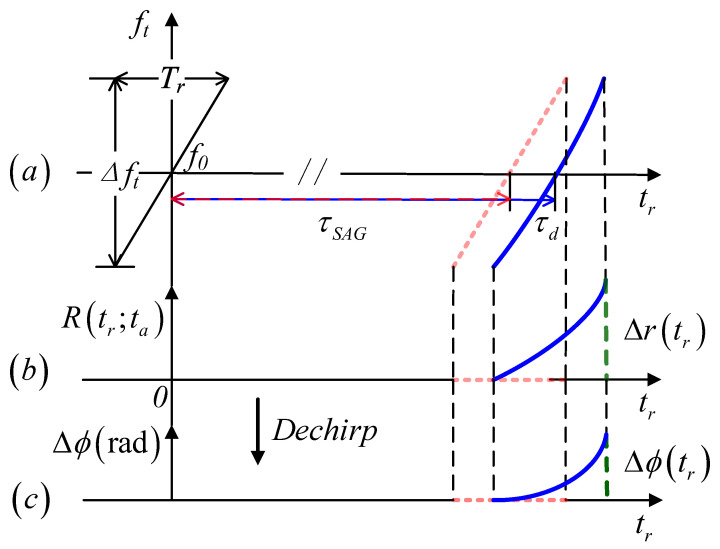
Comparison of the signal models between the “stop-and-go” assumption and the actual echo model. The black solid line represents the transmitted signal. Red dashed lines represent the echo model under the “stop-and-go” assumption. Blue solid lines represent the actual echo model. (**a**) Time–frequency relationship of the transmitted and received signals, where $\tau_{SAG}$ and $\tau_{d}$ represent the propagation delays of both models. $\Delta f_{t}$ represents the pulse bandwidth. $T_{r}$ represents the pulse duration. $f_{0}$ represents the initial frequency. (**b**) Bistatic range variation during the sweep, where $\Delta r(t_{r})$ represents the range deviation. (**c**) Residual phase error after dechirp, where $\Delta \phi (t_{r})$ represents the phase error.

To further elucidate the physical mechanism underlying the breakdown of the “stop-and-go” assumption for the high-maneuvering airborne receiver, Figure 3 illustrates a comparison of the signal characteristics between the echo model under the “stop-and-go” assumption and the actual echo model within a single sweep duration. As shown in Figure 3a, the “stop-and-go” assumption posits that the slant range remains constant during the sweep and results in a fixed propagation delay $\tau_{SAG}$. However, due to the high maneuverability of the airborne receiver and the long sweep time of the FMCW signal, the actual delay $\tau_{d}$ is greater than $\tau_{SAG}$, as demonstrated in Figure 3b. Figure 3b illustrates the variation of the bistatic range. Compared to the echo model under the “stop-and-go” assumption that neglects intra-pulse motion, the range deviation $\Delta r(t_{r})$ of radar platforms under the actual model varies continuously with the fast time $t_{r}$ and exhibits a nonlinear characteristic due to the receiver acceleration. Figure 3c illustrates the difference in phase error between the two models after dechirp. Under the “stop-and-go” assumption, no additional phase deviation is introduced within the pulse due to the accelerated motion of the receiver, thereby maintaining a constant beat frequency and yielding zero phase error. However, the actual echo model manifests a nonlinear phase error $\Delta \phi (t_{r})$, which leads to defocusing in range and a geometric shift if its peak value exceeds π/4.

The actual propagation delay $\tau_{d}$ is obtained by solving Equation (4). However, due to the high maneuverability of the airborne receiver and the square root operation in the equation, the range history from the receiver to the target is not merely a function of fast and slow time. Unlike the propagation delay under the “stop-and-go” assumption in Equation (3), the actual propagation delay in Equation (4) is defined by a self-referential higher-order function, which means that there exists no exact analytical expression for this equation.

To simplify the calculation, we propose the following assumption: the signal duration is discretized into infinitesimal time units, within which the position of the receiver is assumed constant. Consequently, the propagation delay approximation based on the “stop-and-go” assumption is substituted into the quadratic term of the range history of the receiver. The propagation delay approximation term (called ${\tilde{\tau }}_{SAG}$) is expressed as

(7) $${\tilde{\tau }}_{SAG}\left(t_{a},t_{r}\right)=\frac{R_{T}\left(t_{a},t_{r}\right)+R_{R}\left(t_{a},t_{r}\right)}{c}$$

(8) $$R_{R}\left(t_{a},t_{r}\right)=\left|r_{R}+v_{R}\left(t_{a}+t_{r}\right)+a_{R}{\left(t_{a}+t_{r}\right)}^{2}/2-r_{P}\right|$$

where $R_{R}\left(t_{a},t_{r}\right)$ represents the range history between the target and the receiver within one unit. Equation (6) is rewritten as

(9) $$\begin{array}{cc} R_{R}\left(t_{a},t_{r};\tau_{d}\left(t_{a},t_{r}\right)\right) & =\left|r_{R}+v_{R}\left(t_{a}+t_{r}+\tau_{d}\left(t_{a},t_{r}\right)\right)+a_{R}{\left(t_{a}+t_{r}+\tau_{d}\left(t_{a},t_{r}\right)\right)}^{2}/2-r_{P}\right|\\ \; & \approx \left|r_{R}+v_{R}\left(t_{a}+t_{r}+\tau_{d}\left(t_{a},t_{r}\right)\right)+a_{R}{\left(t_{a}+t_{r}+{\tilde{\tau }}_{SAG}\left(t_{a},t_{r}\right)\right)}^{2}/2-r_{P}\right|\\ \; & ={\tilde{R}}_{R}\left(t_{a},t_{r};\tau_{d}\left(t_{a},t_{r}\right)\right) \end{array}$$

By substituting Equation (9) into Equation (4), we obtain

(10) $$\tau_{d}\left(t_{a},t_{r}\right)=\frac{R_{T}\left(t_{a},t_{r}\right)+{\tilde{R}}_{R}\left(t_{a},t_{r};\tau_{d}\left(t_{a},t_{r}\right)\right)}{c}$$

Equation (10) is converted as follows:

(11) $$c\tau_{d}\left(t_{a},t_{r}\right)-R_{T}\left(t_{a},t_{r}\right)={\tilde{R}}_{R}\left(t_{a},t_{r};\tau_{d}\left(t_{a},t_{r}\right)\right)$$

(12) $$c\tau_{d}\left(t_{a},t_{r}\right)-\left|r_{T}+v_{T}\left(t_{a}+t_{r}\right)-r_{P}\right|=\left|\begin{array}{c} r_{R}+v_{R}\left(t_{a}+t_{r}+\tau_{d}\left(t_{a},t_{r}\right)\right)\\ +a_{R}{\left(t_{a}+t_{r}+{\tilde{\tau }}_{SAG}\left(t_{a},t_{r}\right)\right)}^{2}/2-r_{P} \end{array}\right|$$

By squaring both sides of Equation (12), we obtain the following relation:

(13) $$(c\tau_{d}\left(t_{a},t_{r}\right)-|r_{T}+v_{T}\left(t_{a}+t_{r}\right)-r_{P}{\left|\right)}^{2}={\left|\begin{array}{c} r_{R}+v_{R}\left(t_{a}+t_{r}\right)\\ +a_{R}{\left(t_{a}+t_{r}+{\tilde{\tau }}_{SAG}\left(t_{a},t_{r}\right)\right)}^{2}/2\\ -r_{P}+v_{R}\tau_{d}\left(t_{a},t_{r}\right) \end{array}\right|}^{2}$$

Equation (13) is further simplified to the following form:

(14) $$\left(c^{2}-|v_{R}{|}^{2}\right)\tau_{d}^{2}\left(t_{a},t_{r}\right)-2(c|r_{T}\left(t_{a},t_{r}\right)|+r_{R}\left(t_{a},t_{r}\right)\cdot v_{R})\tau_{d}\left(t_{a},t_{r}\right)+\left|r_{T}\left(t_{a},t_{r}\right){|}^{2}-\right|r_{R}\left(t_{a},t_{r}\right){|}^{2}=0$$

where · represents the inner product of two vectors, and

(15) $$r_{T}\left(t_{a},t_{r}\right)=r_{T}+v_{T}\left(t_{a}+t_{r}\right)-r_{P}$$

(16) $$r_{R}\left(t_{a},t_{r}\right)=r_{R}+v_{R}\left(t_{a}+t_{r}\right)+a_{R}{\left(t_{a}+t_{r}+{\tilde{\tau }}_{SAG}\left(t_{a},t_{r}\right)\right)}^{2}/2-r_{P}$$

Note that the discriminant of Equation (14) is greater than zero, indicating that it has two solutions. One of the solutions is an extraneous solution generated by the transformation process from Equation (10) to Equation (14). Following that, the high-precision propagation delay of GEO SA FMCW BiSAR (called $\tau_{HP}$) is obtained by solving Equation (14):

(17) $$\tau_{HP}\left(t_{a},t_{r}\right)=\frac{1}{c^{2}-{\left|v_{R}\right|}^{2}}\left\{\begin{array}{c} c|r_{T}\left(t_{a},t_{r}\right)|+r_{R}\left(t_{a},t_{r}\right)\cdot v_{R}\\ +\sqrt{\begin{array}{c} 2c|r_{T}\left(t_{a},t_{r}\right)|r_{R}\left(t_{a},t_{r}\right)\cdot v_{R}\\ +|r_{R}\left(t_{a},t_{r}\right)\cdot v_{R}{|}^{2}+c^{2}{\left|r_{R}\left(t_{a},t_{r}\right)\right|}^{2}\\ +|v_{R}{|}^{2}\left(\right|r_{T}\left(t_{a},t_{r}\right){|}^{2}-|r_{R}\left(t_{a},t_{r}\right){|}^{2}) \end{array}} \end{array}\right\}$$

By incorporating the propagation delay formula presented in Equation (17), the proposed echo model for the high-maneuvering airborne receiver is expressed as

(18) $$s_{r}\left(t_{a},t_{r}\right)=w_{a}\left(\frac{t_{a}-t_{a0}}{T_{a}}\right)w_{r}\left(\frac{t_{r}-\tau_{HP}\left(t_{a},t_{r}\right)}{T_{r}}\right)\exp\left\{j2\pi f_{0}\left(t_{r}-\tau_{HP}\left(t_{a},t_{r}\right)\right)\right\}\exp\left\{j\pi K_{r}{\left(t_{r}-\tau_{HP}\left(t_{a},t_{r}\right)\right)}^{2}\right\}$$

where $w_{r}\left(\cdot \right)$ and $w_{a}\left(\cdot \right)$ are the window functions for the range and azimuth directions, respectively. In this paper, both are assumed to be rectangular windows; $T_{a}$ and $T_{r}$ are the synthetic aperture time and the pulse width of the FMCW signal, respectively; $t_{a0}$ is the Doppler center shift time caused by the equivalent squint angle in the transmit–receive azimuth direction; $f_{0}$ is the radar operating center frequency; $K_{r}$ is the chirp modulation rate.

### 2.3. Echo Characteristic Analysis

To assess the proposed echo model, time delay error (TDE) and quadratic phase error (QPE) are employed as metrics for quantitative accuracy analysis. Both the proposed echo model and the echo model under the “stop-and-go” assumption are compared against the actual received signal. The simulation parameters are presented in Table 3.

The equations for calculating TDE are as follows:

(19) $$TDE_{\mathrm{the}\;\mathrm{proposed}\;\mathrm{model}}\left(t\right)=\left|\tau_{HP}\left(t_{a},t_{r}\right)-\tau_{d}\left(t_{a},t_{r}\right)\right|$$

(20) $$TDE_{“\mathrm{stop-and-go}”\;\mathrm{model}}\left(t\right)=\left|\tau_{SAG}\left(t_{a},t_{r}\right)-\tau_{d}\left(t_{a},t_{r}\right)\right|$$

where *t* represents the global observation time, which is related to the fast time and slow time variables by $t=t_{r}+t_{a}$. $\tau_{d}\left(t_{a},t_{r}\right)$ denotes the actual echo propagation delay of GEO SA FMCW BiSAR with the high-maneuvering receiver. Its numerical solution is computed by solving Equation (4). $\tau_{SAG}\left(t_{a},t_{r}\right)$ is obtained from Equation (3), and $\tau_{HP}\left(t_{a},t_{r}\right)$ is obtained from Equation (17). Simultaneously, QPE is defined as follows:

(21) $$QPE_{\mathrm{the}\;\mathrm{proposed}\;\mathrm{model}}=\pi f_{0}{\left|\frac{\partial^{2}\tau_{HP}\left(t_{a},t_{r}\right)}{\partial t^{2}}-\frac{\partial^{2}\tau_{d}\left(t_{a},t_{r}\right)}{\partial t^{2}}\right|}_{t=0}{\left(\frac{T_{a}}{2}\right)}^{2}$$

(22) $$QPE_{“\mathrm{stop-and-go}”\;\mathrm{model}}=\pi f_{0}{\left|\frac{\partial^{2}\tau_{SAG}\left(t_{a},t_{r}\right)}{\partial t^{2}}-\frac{\partial^{2}\tau_{d}\left(t_{a},t_{r}\right)}{\partial t^{2}}\right|}_{t=0}{\left(\frac{T_{a}}{2}\right)}^{2}$$

**Figure 4:**
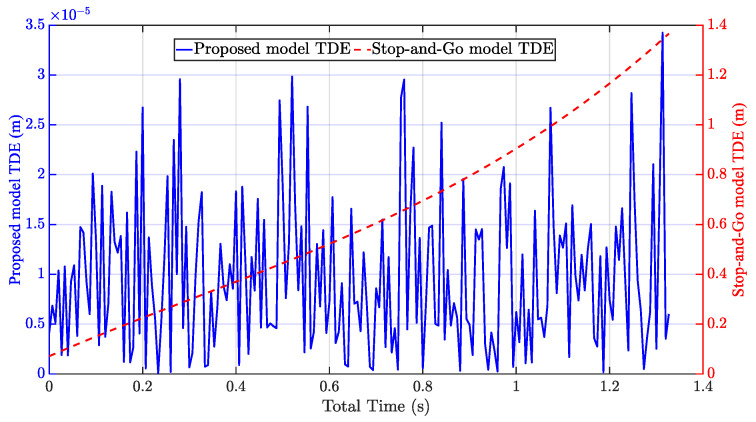
TDE of echoes in the GEO SA FMCW BiSAR system: Comparison between the proposed echo model and the echo model under the “stop-and-go” assumption.

To analyze the magnitude of the range walk induced by TDE, we evaluate it in meters. Figure 4 compares the TDE of different models with the actual received echo. The result shows that during the global observation time, the TDE between the proposed echo model and the actual echo is on the order of $\times 10^{-5}$ meters, which is almost negligible. In contrast, the peak value of the TDE of the echo model under the “stop-and-go” assumption exceeds 1.4 m. Such an error leads to severe range offset and defocusing of the target in the final image.

**Figure 5:**
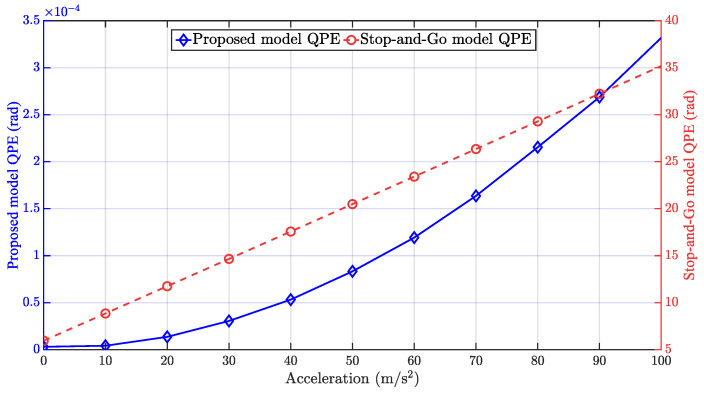
QPE regarding the vertical acceleration in the GEO SA FMCW BiSAR system: Comparison between the proposed echo model and the echo model under the “stop-and-go” assumption.

To evaluate QPE, the acceleration direction is set along the vertical direction, with acceleration values ranging from 0 to 100 $\;m/s^{2}$. As shown in Figure 5, as the acceleration changes, the QPE of the proposed model remains within $3.5\times 10^{-4}$ rad throughout the tested acceleration range, and this error level is less than π/4 rad, which is negligible for final image focusing. However, the QPE of the echo model under the “stop-and-go” assumption is as high as 35 rad, and such an error leads to azimuth defocusing.

**Figure 6:**
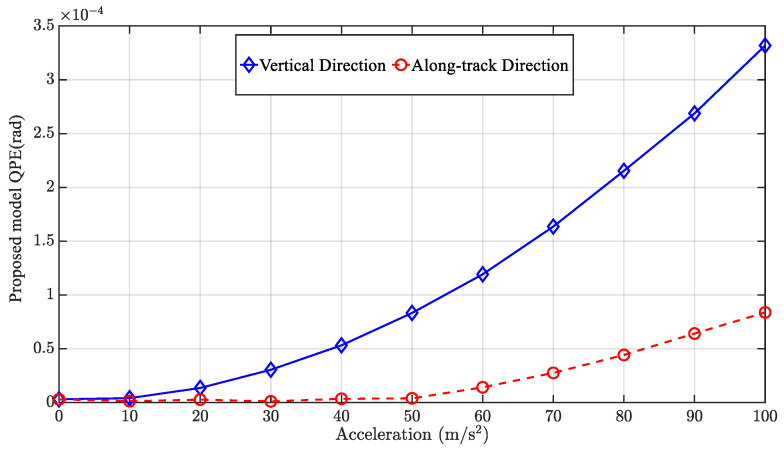
Comparison of QPE results between the vertical direction acceleration and the along-track direction acceleration

Additionally, the sensitivity of the QPE to the receiver acceleration direction is investigated by comparing the vertical and along-track acceleration cases. The acceleration vectors are defined as $a_{R}=\left(0,0,a_{z}\right)$ and $a_{R}=\left(0,a_{y},0\right)$, respectively. For a fair comparison, the acceleration values are varied from 0 to 100 $\;m/s^{2}$. As illustrated in Figure 6, the QPE results for both directional accelerations remain on the order of $10^{-4}$ rad, which is far below the threshold of π/4 rad. This demonstrates the accuracy of the proposed model across different maneuvering directions. Meanwhile, the QPE induced by vertical acceleration is higher than that of along-track acceleration (approximately four times larger at 100 $\;m/s^{2}$). This result confirms that the GEO SA FMCW BiSAR is more sensitive to vertical maneuvers. Therefore, we validate the proposed echo model by focusing on the vertical acceleration.

## 3. The 2-D Frequency-Domain Imaging Algorithm

In the GEO SA FMCW BiSAR system, the received signal incorporates the motion of the receiver during the propagation delay, while the coupling between slow-time and fast-time becomes more pronounced. Additionally, traditional time-domain imaging algorithms based on point-by-point matched filtering exhibit low efficiency. Therefore, this section proposes an efficient 2-D frequency-domain imaging algorithm through echo model analysis for GEO SA FMCW BiSAR with the high-maneuvering airborne receiver.

### 3.1. Derivation of Imaging Algorithm

The propagation delay equation in the proposed model is excessively complex. Therefore, for computational efficiency, $\tau_{HP}$ is approximated by a fourth-order Taylor-series expansion with respect to the slow-time $t_{a}$ around the aperture center ($t_{a}=0$), which is expanded as

(23) $$\tau_{HP}^{\left(4\right)}\left(t_{a},t_{r}\right)=\alpha_{0}+\alpha_{1}t_{a}+\alpha_{2}{t_{a}}^{2}+\alpha_{3}{t_{a}}^{3}+\alpha_{4}{t_{a}}^{4}$$

where the coefficients of the fourth-order Taylor expansion are

(24) $$\begin{array}{cc} \; & \alpha_{0}={\left.\tau_{HP}\left(t_{a},t_{r}\right)\right|}_{t_{a}=0}\\ \; & \alpha_{1}={\left.\frac{\partial \tau_{HP}\left(t_{a},t_{r}\right)}{\partial t_{a}}\right|}_{t_{a}=0}\\ \; & \alpha_{2}={\left.\frac{1}{2!}\frac{\partial^{2}\tau_{HP}\left(t_{a},t_{r}\right)}{\partial {t_{a}}^{2}}\right|}_{t_{a}=0}\\ \; & \alpha_{3}={\left.\frac{1}{3!}\frac{\partial^{3}\tau_{HP}\left(t_{a},t_{r}\right)}{\partial {t_{a}}^{3}}\right|}_{t_{a}=0}\\ \; & \alpha_{4}=\frac{1}{4!}\frac{\partial^{4}\tau_{HP}\left(t_{a},t_{r}\right)}{\partial {t_{a}}^{4}}{|}_{t_{a}=0} \end{array}$$

The obtained fourth-order Taylor polynomial is then substituted into Equation (18). Consequently, we have

(25) $$\begin{array}{c} \begin{array}{cc} s_{r}\left(t_{a},t_{r}\right) & =w_{a}\left(\frac{t_{a}-t_{a0}}{T_{a}}\right)w_{r}\left(\frac{t_{r}-\tau_{HP}^{\left(4\right)}\left(t_{a},t_{r}\right)}{T_{r}}\right)\\ \; & \;\exp\left\{j2\pi f_{0}\left(t_{r}-\tau_{HP}^{\left(4\right)}\left(t_{a},t_{r}\right)\right)\right\}\exp\left\{j\pi K_{r}{\left(t_{r}-\tau_{HP}^{\left(4\right)}\left(t_{a},t_{r}\right)\right)}^{2}\right\} \end{array} \end{array}$$

After dechirp, we obtain

(26) $$\begin{array}{cc} s_{if}\left(t_{a},t_{r}\right) & =s_{r}\left(t_{a},t_{r}\right)H_{0}=s_{r}\left(t_{a},t_{r}\right)s_{ref}^{*}\left(t_{a},t_{r}\right)\\ \; & =w_{a}\left(\frac{t_{a}-t_{a0}}{T_{a}}\right)w_{r}\left(\frac{t_{r}-\tau_{HP}^{\left(4\right)}\left(t_{a},t_{r}\right)}{T_{r}}\right)\\ \; & \;\exp\left\{-j2\pi K_{r}\left(t_{r}-R_{ref}/c\right)\left(\tau_{HP}^{\left(4\right)}\left(t_{a},t_{r}\right)-R_{ref}/c\right)\right\}\\ \; & \;\exp\left\{-j2\pi f_{0}\left(\tau_{HP}^{\left(4\right)}\left(t_{a},t_{r}\right)-R_{ref}/c\right)\right\}\\ \; & \;\exp\left\{j\pi K_{r}{\left(\tau_{HP}^{\left(4\right)}\left(t_{a},t_{r}\right)-R_{ref}/c\right)}^{2}\right\} \end{array}$$

and the reference signal for the dechirp operation is expressed as

(27) $$\begin{array}{c} \begin{array}{cc} H_{0} & =s_{ref}^{*}\left(t_{a},t_{r}\right)\\ \; & =\exp\left\{-j2\pi f_{0}\left(t_{r}-R_{ref}/c\right)\right\}\exp\left\{-j\pi K_{r}{\left(t_{r}-R_{ref}/c\right)}^{2}\right\} \end{array} \end{array}$$

where ${\left(\cdot \right)}^{*}$ denotes the conjugate operation and $R_{ref}$ denotes the minimum bistatic range between the radar platforms and the target.

In Equation (26), the first exponential term corresponds to the range information; its Fourier transform along the range dimension produces the range compression result. The second exponential term contains the azimuth phase history required for azimuth-matched filtering. The third exponential term represents the RVP effect induced during the dechirp operation. The RVP term does not provide useful information but rather introduces complications into the subsequent imaging process. Consequently, its removal is essential prior to imaging. The compensation equation for removing RVP is

(28) $$H_{1}=\exp\left\{-j\pi K_{r}{\left(\tau_{HP}^{\left(4\right)}\left(t_{a},t_{r}\right)-R_{ref}/c\right)}^{2}\right\}$$

After compensating for the RVP term, the echo signal after dechirp is converted to:

(29) $$\begin{array}{cc} s_{0}\left(t_{a},t_{r}\right) & =w_{a}\left(\frac{t_{a}-t_{a0}}{T_{a}}\right)w_{r}\left(\frac{t_{r}-\tau_{HP}^{\left(4\right)}\left(t_{a},t_{r}\right)}{T_{r}}\right)\\ \; & \;\exp\left\{-j2\pi K_{r}\left(t_{r}-R_{ref}/c\right)\left(\tau_{HP}^{\left(4\right)}\left(t_{a},t_{r}\right)-R_{ref}/c\right)\right\}\\ \; & \;\exp\left\{-j2\pi f_{0}\left(\tau_{HP}^{\left(4\right)}\left(t_{a},t_{r}\right)-R_{ref}/c\right)\right\} \end{array}$$

After the aforementioned dechirp operation (time–frequency transformation), the time-domain echo signal is transformed into the range spectrum domain. Since the range frequency is symmetric about the origin, the relationship between range and frequency in the range direction is expressed as

(30) $$f_{r}=K_{r}\left(t_{r}-R_{ref}/c\right)$$

Substituting Equation (30) into Equation (29), we get:

(31) $$s_{0}\left(t_{a},f_{r}\right)=w_{a}\left(\frac{t_{a}-t_{a0}}{T_{a}}\right)W_{r}\left(f_{r}\right)\exp\left\{-j2\pi \left(f_{r}+f_{0}\right)\left(\tau_{HP}^{\left(4\right)}\left(t_{a},t_{r}\right)-R_{ref}/c\right)\right\}$$

where $W_{r}\left(f_{r}\right)$ denotes the range–frequency envelope obtained from the Fourier transform of the range window $w_{r}$. To convert the echo signal of GEO SA FMCW BiSAR into the 2-D frequency-domain, an azimuth Fourier transform needs to be applied to $s_{0}\left(t_{a},f_{r}\right)$. Specifically, MSR is utilized to determine the azimuth stationary phase point, thereby yielding the 2-D spectrum. Substituting Equation (23) into Equation (31), the echo signal phase of GEO SA FMCW BiSAR is written as

(32) $$\varphi \left(t_{a},f_{r}\right)=-2\pi \left(f_{r}+f_{0}\right)\left(\alpha_{0}+\alpha_{1}t_{a}+\alpha_{2}t_{a}^{2}+\alpha_{3}t_{a}^{3}+\alpha_{4}t_{a}^{4}-R_{ref}/c\right)$$

To simplify the application of MSR, the linear component is removed from the phase in Equation (32). Thereby, the echo signal phase of GEO SA FMCW BiSAR is rewritten as

(33) $$\varphi \left(t_{a},f_{r}\right)=-2\pi \left(f_{r}+f_{0}\right)\left(\alpha_{0}+\alpha_{2}t_{a}^{2}+\alpha_{3}t_{a}^{3}+\alpha_{4}t_{a}^{4}-R_{ref}/c\right)$$

By applying the principle of stationary phase (POSP), we obtain the mapping relationship between the slow-time $t_{a}$ and the azimuth frequency $f_{a}$, which is expressed as

(34) $$\phi \left(t_{a},f_{r}\right)=-2\pi \left(f_{r}+f_{0}\right)\left(\alpha_{0}+\alpha_{2}t_{a}^{2}+\alpha_{3}t_{a}^{3}+\alpha_{4}t_{a}^{4}-R_{ref}/c\right)-2\pi f_{a}t_{a}$$

Finding the partial derivative of Equation (34) with respect to $t_{a}$:

(35) $$\frac{\partial \phi (t_{a},f_{r})}{\partial t_{a}}=-2\pi \left(f_{r}+f_{0}\right)\left(2\alpha_{2}t_{a}+3\alpha_{3}t_{a}^{2}+4\alpha_{4}t_{a}^{3}\right)-2\pi f_{a}$$

By setting Equation (35) to zero, the relationship between time and frequency in the azimuth direction is expressed as

(36) $$-\frac{f_{a}}{f_{r}+f_{0}}=2\alpha_{2}t_{a}+3\alpha_{3}t_{a}^{2}+4\alpha_{4}t_{a}^{3}$$

Through MSR, we obtain the stationary phase point in the azimuth direction, which is expressed as

(37) $$t_{a}\left(f_{a}\right)=-\frac{f_{a}}{2\alpha_{2}\left(f_{r}+f_{0}\right)}-\frac{3\alpha_{3}f_{a}^{2}}{8\alpha_{2}^{3}{\left(f_{r}+f_{0}\right)}^{2}}-\frac{\left(9\alpha_{3}^{2}-4\alpha_{2}\alpha_{4}\right)f_{a}^{3}}{16\alpha_{2}^{5}{\left(f_{r}+f_{0}\right)}^{3}}$$

Substituting Equation (37) into Equation (34), the 2-D frequency-domain phase of the echo signal is expressed as

(38) $$\begin{array}{cc} \phi (f_{a},f_{r}) & =-2\pi \left(f_{r}+f_{0}\right)\left(\alpha_{0}+\alpha_{2}t_{a}^{2}\left(f_{a}\right)+\alpha_{3}t_{a}^{3}\left(f_{a}\right)+\alpha_{4}t_{a}^{4}\left(f_{a}\right)\right.\left.-R_{ref}/c\right)\\ \; & \;-2\pi f_{a}t_{a}\left(f_{a}\right) \end{array}$$

Notably, since the linear component of the echo signal was removed prior to the application of POSP, it needs to be compensated at this stage. Based on Fourier transform properties, equivalent compensation for this linear component is achieved by introducing a frequency shift to the 2-D frequency-domain signal, i.e.,

(39) $$s_{0}\left(t_{a},f_{r}\right)\exp\left\{-j2\pi \left(f_{r}+f_{0}\right)\alpha_{1}t_{a}\right\}\overset{F_{t_{a}}}{\to }S_{0}\left(f_{a}+\left(f_{r}+f_{0}\right)\alpha_{1},f_{r}\right)$$

and $t_{a}\left(f_{a}\right)$ is transformed into $t_{a}\left(f_{a}+\left(f_{r}+f_{0}\right)\alpha_{1}\right)$. Accordingly, Equation (37) is rewritten as

(40) $$t_{a}\left(f_{a}+\left(f_{r}+f_{0}\right)\alpha_{1}\right)=-\frac{f_{a}+\left(f_{r}+f_{0}\right)\alpha_{1}}{2\alpha_{2}\left(f_{r}+f_{0}\right)}-\frac{3\alpha_{3}{\left(f_{a}+\left(f_{r}+f_{0}\right)\alpha_{1}\right)}^{2}}{8\alpha_{2}^{3}{\left(f_{r}+f_{0}\right)}^{2}}-\frac{\left(9\alpha_{3}^{2}-4\alpha_{2}\alpha_{4}\right){\left(f_{a}+\left(f_{r}+f_{0}\right)\alpha_{1}\right)}^{3}}{16\alpha_{2}^{5}{\left(f_{r}+f_{0}\right)}^{3}}$$

Thereby, the 2-D spectrum expression of GEO SA FMCW BiSAR with the high-maneuvering receiver is expressed as

(41) $$S_{0}\left(f_{a},f_{r}\right)=W_{a}\left(f_{a}-f_{a0}\right)W_{r}\left(f_{r}\right)\exp\left\{j\phi (f_{a},f_{r})\right\}$$

where $W_{a}\left(f_{a}-f_{a0}\right)$ denotes the azimuth-frequency envelope, $f_{a0}$ denotes the azimuth Doppler center frequency, and $\phi (f_{a},f_{r})$ represents the 2-D frequency-domain phase, with the latter expressed as follows:

(42) $$\phi \left(f_{a},f_{r}\right)=-2\pi \left(f_{r}+f_{0}\right)\left(\begin{array}{c} \alpha_{0}\\ +\alpha_{2}t_{a}^{2}\left(f_{a}+\left(f_{r}+f_{0}\right)\alpha_{1}\right)\\ +\alpha_{3}t_{a}^{3}\left(f_{a}+\left(f_{r}+f_{0}\right)\alpha_{1}\right)\\ +\alpha_{4}t_{a}^{4}\left(f_{a}+\left(f_{r}+f_{0}\right)\alpha_{1}\right)\\ -R_{ref}/c \end{array}\right)-2\pi f_{a}t_{a}\left(f_{a}+\left(f_{r}+f_{0}\right)\alpha_{1}\right)$$

To decouple the 2-D spectrum between the range and azimuth directions, we compensate for the residual phase in Equation (41).

(43) $$H_{2}=\exp\left\{-j\phi (f_{a},f_{r})\right\}$$

Following the residual-phase compensation, the 2-D inverse Fourier transform is applied to the 2-D spectrum to obtain the focused image.

(44) $$image\left(t_{a},t_{r}\right)=\mathrm{IFFT}\left\{W_{a}\left(f_{a}-f_{a0}\right)W_{r}\left(f_{r}\right)\right\}=sinc\left(t_{a}-t_{a0}\right)sinc\left(t_{r}\right)$$

**Figure 7:**
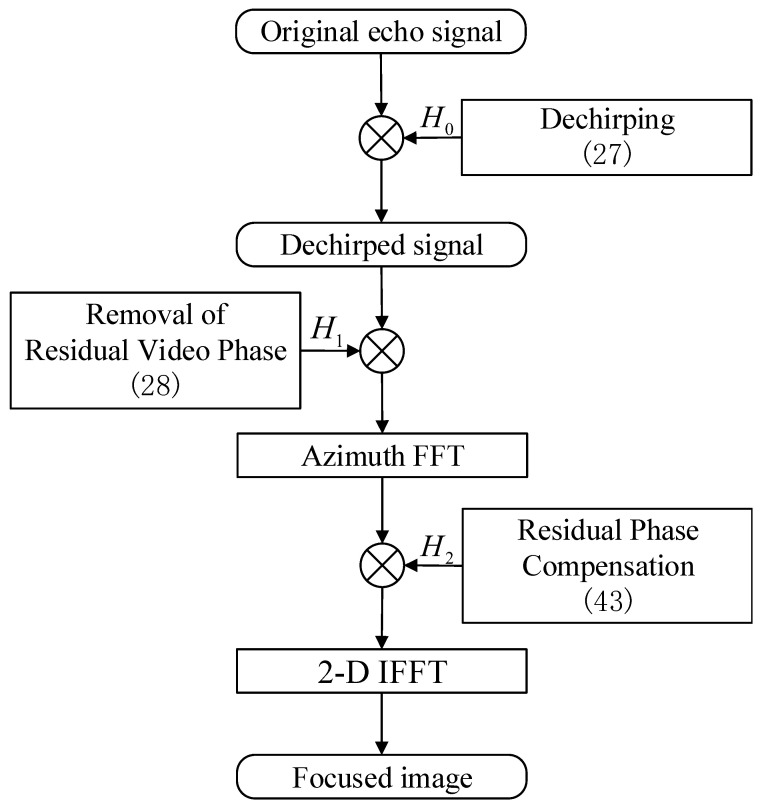
Process of 2-D frequency-domain imaging algorithm.

This algorithmic flow is illustrated in Figure 7.

### 3.2. Algorithm Performance Analysis

To validate the feasibility of the proposed algorithm, we perform a third-order Taylor expansion of the residual phase. This analysis demonstrates that the subsequent phase compensation simultaneously achieves azimuth compression, RCMC, SRC, and high-order range–azimuth decoupling.

Using a third-order Taylor expansion of Equation (42) with respect to $f_{r}$, we obtain

(45) $$\phi \left(f_{a},f_{r}\right)\approx \omega_{0}\left(f_{a}\right)+\omega_{1}\left(f_{a}\right)f_{r}+\omega_{2}\left(f_{a}\right)f_{r}^{2}+\omega_{3}\left(f_{a}\right)f_{r}^{3}$$

The first term of Equation (45), solely dependent on azimuth frequency, constitutes the azimuth modulation term. Its compensation achieves azimuth compression. The second term, linear with respect to range frequency, corresponds to range migration. Compensating for it achieves RCMC. The third term, quadratic with respect to range frequency, is the residual range modulation term. Its compensation achieves SRC. The fourth term represents the high-order range–azimuth coupling term, resulting from the interaction between slow-time and fast-time. Compensating for this term achieves high-order decoupling. The coefficients of the third-order Taylor expansion are as follows:

(46) $$\begin{array}{cc} \; & \omega_{0}\left(f_{a}\right)=2\pi f_{a}\mu_{0}-2\pi f_{0}\left(\alpha_{0}+\alpha_{2}\mu_{0}^{2}-\alpha_{3}\mu_{0}^{3}+\alpha_{4}\mu_{0}^{4}-R_{ref}/c\right)\\ \; & \omega_{1}\left(f_{a}\right)=-2\pi \left(\alpha_{0}-f_{0}\mu_{1}\left(4\alpha_{4}\mu_{0}^{3}-3\alpha_{3}\mu_{0}^{2}+2\alpha_{2}\mu_{0}\right)+\alpha_{2}\mu_{0}^{2}-\alpha_{3}\mu_{0}^{3}+\alpha_{4}\mu_{0}^{4}-R_{ref}/c+f_{a}\mu_{1}\right)\\ \; & \omega_{2}\left(f_{a}\right)=2\pi \left\{\begin{array}{c} f_{a}\mu_{2}-f_{0}\left(\begin{array}{c} \alpha_{2}\left(\mu_{1}^{2}+2\mu_{2}\mu_{0}\right)\\ -\alpha_{3}\left(\left(\mu_{1}^{2}+2\mu_{2}\mu_{0}\right)\mu_{0}+\mu_{2}\mu_{0}^{2}+2\mu_{0}\mu_{1}^{2}\right)\\ +\alpha_{4}\left(4\mu_{0}^{2}\mu_{1}^{2}+2\left(\mu_{1}^{2}+2\mu_{2}\mu_{0}\right)\mu_{0}^{2}\right) \end{array}\right)-3\alpha_{3}\mu_{0}^{2}\mu_{1}+4\alpha_{4}\mu_{0}^{3}\mu_{1}+2\alpha_{2}\mu_{0}\mu_{1} \end{array}\right\}\\ \; & \omega_{3}\left(f_{a}\right)=-2\pi \left\{\begin{array}{c} \alpha_{2}\left(\mu_{1}^{2}+2\mu_{2}\mu_{0}\right)\alpha_{3}\left(\left(\mu_{1}^{2}+2\mu_{2}\mu_{0}\right)\mu_{0}+\mu_{2}\mu_{0}^{2}+2\mu_{0}\mu_{1}^{2}\right)\\ +f_{a}\mu_{3}+\alpha_{4}\left(4\mu_{0}^{2}\mu_{1}^{2}+2\left(\mu_{1}^{2}+2\mu_{2}\mu_{0}\right)\mu_{0}^{2}\right)\\ -f_{0}\left(\begin{array}{c} \alpha_{4}\left(2\left(2\mu_{2}\mu_{1}+2\mu_{0}\mu_{3}\right)\mu_{0}^{2}+4\left(\mu_{1}^{2}+2\mu_{2}\mu_{0}\right)\mu_{0}\mu_{1}\right)\\ -\alpha_{3}\left(\begin{array}{c} \mu_{0}^{2}\mu_{3}+\left(2\mu_{2}\mu_{1}+2\mu_{0}\mu_{3}\right)\mu_{0}\\ +\left(\mu_{1}^{2}+2\mu_{2}\mu_{0}\right)\mu_{1}+2\mu_{2}\mu_{0}\mu_{1} \end{array}\right)\\ +\alpha_{2}\left(2\mu_{2}\mu_{1}+2\mu_{0}\mu_{3}\right) \end{array}\right) \end{array}\right\} \end{array}$$

The coefficients $\mu_{0}$, $\mu_{1}$, $\mu_{2}$, and $\mu_{3}$ in the above Taylor series are shown below:

(47) $$\begin{array}{cc} \; & \mu_{0}=\frac{f_{a}+\alpha_{1}f_{0}}{2\alpha_{2}f_{0}}+\frac{3\alpha_{3}{\left(f_{a}+\alpha_{1}f_{0}\right)}^{2}}{8\alpha_{2}^{3}f_{0}^{2}}+\frac{\left(9\alpha_{3}^{2}-4\alpha_{2}\alpha_{4}\right){\left(f_{a}+\alpha_{1}f_{0}\right)}^{3}}{16\alpha_{2}^{5}f_{0}^{3}}\\ \; & \mu_{1}=\frac{f_{a}}{2\alpha_{2}f_{0}^{2}}+\frac{3\alpha_{3}\left({\left(f_{a}+\alpha_{1}f_{0}\right)}^{2}-\alpha_{1}f_{0}\left(f_{a}+\alpha_{1}f_{0}\right)\right)}{4\alpha_{2}^{3}f_{0}^{3}}\\ \; & \;+\frac{3\left(9\alpha_{3}^{2}-4\alpha_{2}\alpha_{4}\right)\left({\left(f_{a}+\alpha_{1}f_{0}\right)}^{3}-\alpha_{1}f_{0}{\left(f_{a}+\alpha_{1}f_{0}\right)}^{2}\right)}{16\alpha_{2}^{5}f_{0}^{4}}\\ \; & \mu_{2}=\frac{f_{a}}{2\alpha_{2}f_{0}^{3}}+\frac{3\alpha_{3}\left(3{\left(f_{a}+\alpha_{1}f_{0}\right)}^{2}+\alpha_{1}^{2}f_{0}^{2}-4\alpha_{1}f_{0}\left(f_{a}+\alpha_{1}f_{0}\right)\right)}{8\alpha_{2}^{3}f_{0}^{4}}\\ \; & \;+\frac{3\left(9\alpha_{3}^{2}-4\alpha_{2}\alpha_{4}\right)\left(\begin{array}{c} 2{\left(f_{a}+\alpha_{1}f_{0}\right)}^{3}+\alpha_{1}^{2}f_{0}^{2}\left(f_{a}+\alpha_{1}f_{0}\right)\\ -3\alpha_{1}f_{0}{\left(f_{a}+\alpha_{1}f_{0}\right)}^{2} \end{array}\right)}{16\alpha_{2}^{5}f_{0}^{5}}\\ \; & \mu_{3}=\frac{f_{a}}{2\alpha_{2}f_{0}^{4}}+\frac{3\alpha_{3}\left(2{\left(f_{a}+\alpha_{1}f_{0}\right)}^{2}+\alpha_{1}^{2}f_{0}^{2}-3\alpha_{1}f_{0}\left(f_{a}+\alpha_{1}f_{0}\right)\right)}{4\alpha_{2}^{3}f_{0}^{5}}\\ \; & \;+\frac{\left(9\alpha_{3}^{2}-4\alpha_{2}\alpha_{4}\right)\left(\begin{array}{c} 10{\left(f_{a}+\alpha_{1}f_{0}\right)}^{3}-\alpha_{1}^{3}f_{0}^{3}\\ +9\alpha_{1}^{2}f_{0}^{2}\left(f_{a}+\alpha_{1}f_{0}\right)-18\alpha_{1}f_{0}{\left(f_{a}+\alpha_{1}f_{0}\right)}^{2} \end{array}\right)}{16\alpha_{2}^{5}f_{0}^{5}} \end{array}$$

## 4. Simulation Verification

**Figure 8:**
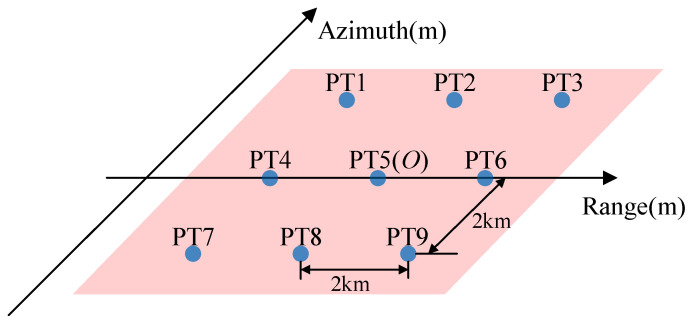
The distribution of point targets.

This section validates the effectiveness of the proposed 2-D frequency-domain imaging algorithm. The system parameters are summarized in Table 3, which is introduced in Section 2.3. The simulation scene is set as shown in Figure 8, where target points are equally spaced in the range and azimuth directions, with a spacing of approximately 2 km. PT5(O) is the central reference target.

To quantitatively evaluate the impact of the echo model under the “stop-and-go” assumption for a high-maneuvering airborne receiver, we analyze the mean squared error (MSE) of the focused images from two echo models. This analysis encompasses a range of initial velocities and accelerations, with a fixed synthetic aperture length (L = 1000 m).

**Figure 9:**
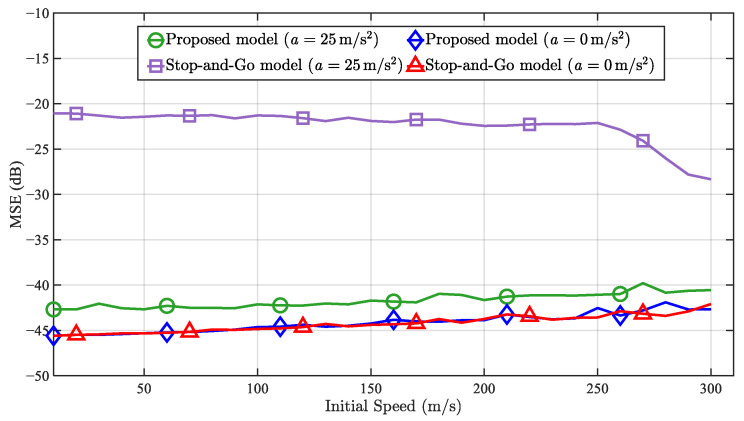
Comparison of MSE variation with initial velocity between the proposed echo model and the echo model under the “stop-and-go” assumption under different motion states.

As illustrated in Figure 9, when a = 0 $m/s^{2}$, the MSE curve of the proposed echo model overlaps with that under the “stop-and-go” assumption. This consistency is expected because the proposed model reduces to the “stop-and-go” model under constant-velocity conditions. This result validates the correctness of our derivation. Subsequently, acceleration (a = 25 $m/s^{2}$) is introduced to simulate a high-maneuvering airborne receiver. The echo model under the “stop-and-go” assumption exhibits a significant error jump under this condition, with its MSE ranging from −28 dB to −21 dB. In contrast, the proposed model demonstrates validity, with its MSE showing only a slight increase and stabilizing between −43 dB and −40 dB. Across the entire velocity range, the proposed model provides an imaging accuracy gain of approximately 15 dB to 20 dB compared to the echo model under the “stop-and-go” assumption. Notably, under the condition of a = 25 $m/s^{2}$, the MSE curve of the echo model under the “stop-and-go” assumption displays a non-monotonic trend, decreasing at higher initial velocities. This is attributed to the fact that for a fixed synthetic aperture length, a higher initial velocity reduces the integration time of the synthetic aperture, which in turn diminishes the cumulative phase error caused by acceleration.

**Figure 10:**
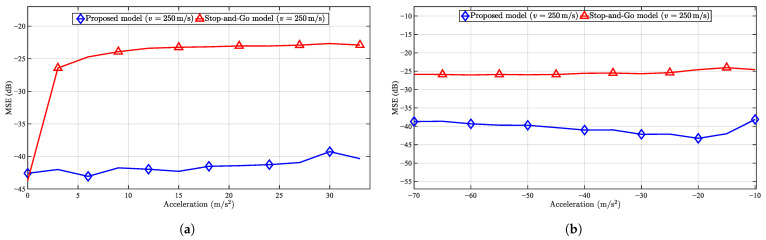
Comparison of MSE at the same initial velocity between the proposed echo model and the echo model under the “stop-and-go” assumption under different accelerations. (**a**) Acceleration is greater than zero. (**b**) Acceleration is less than zero.

Then, to validate the robustness of the proposed model with the high-maneuvering airborne receiver, we examine the impact of platform acceleration on imaging accuracy while keeping the initial velocity fixed. The acceleration direction is set along the vertical direction. Therefore, when the acceleration is positive (a > 0), the receiver is undergoing an accelerating ascent. Conversely, when the acceleration is negative (a < 0), the receiver is undergoing an accelerating descent. As shown in Figure 10a,b, the MSE of the proposed model remains consistently low (below −35 dB), demonstrating its robustness under high-maneuvering motions. In contrast, the MSE of the model under the “stop-and-go” assumption exhibits a significant jump (approx −25 dB) upon the introduction of acceleration and remains at a high level thereafter.

### 4.1. Simulation Results for the Target

**Figure 11:**
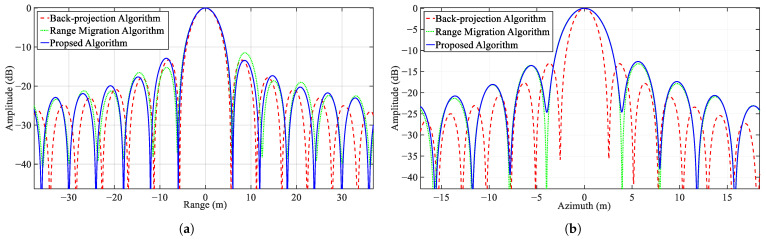
The comparative imaging results of PT5. (**a**) The range profile. (**b**) The azimuth slice profiles.

To verify the effectiveness of the proposed algorithm, the back-projection algorithm, and the range migration algorithm are used to image the central target (PT5) shown in Figure 8. Figure 11 shows the imaging results for all algorithms, respectively.

In the point-target slice profiles, the focusing performance of the proposed algorithm demonstrates comparable quality to both the back-projection algorithm and the range migration algorithm, confirming its effectiveness. We quantitatively evaluate the focusing precision using range and azimuth slice profiles by calculating point-target parameters: peak side-lobe ratio (PSLR), integrated side-lobe ratio (ISLR), and impulse response width (IRW). As shown in Table 4, the range-focusing quality of the proposed algorithm is comparable to that of the back-projection algorithm and superior to that of the range migration algorithm. The slight degradation in azimuth focusing performance of the proposed algorithm is attributed to the fourth-order Taylor-series approximation employed during the MSR process. Furthermore, the proposed algorithm requires 0.229147 s, achieving a speed-up factor of approximately 2.5 compared with the range migration algorithm and more than 570,000 compared with the back-projection algorithm. In summary, the proposed algorithm strikes a balance between computational efficiency and imaging precision, outperforming the time-domain algorithm in speed and the frequency-domain algorithm in accuracy.

**Figure 12:**
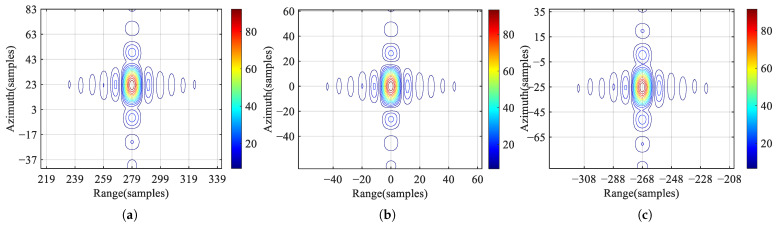
The 2-D frequency-domain algorithm processing for 2-D contour maps of PT3, PT5, and PT7. (**a**) PT3 (**b**) PT5 (**c**) PT7.

Next, to better analyze imaging performance, we perform interpolated magnification on selected point targets PT3, PT5, and PT7, as shown in Figure 12, obtaining contour plots for each target. Point-target evaluation metrics are listed in Table 5.

The theoretical values for PSLR and ISLR are −13.26 dB and −9.6 dB, respectively. As shown in Table 5, both range and azimuth PSLR measurements for PT3, PT5, and PT7 closely match theoretical values. Similarly, ISLR measurements in both dimensions demonstrate close agreement with theoretical expectations.

**Figure 13:**
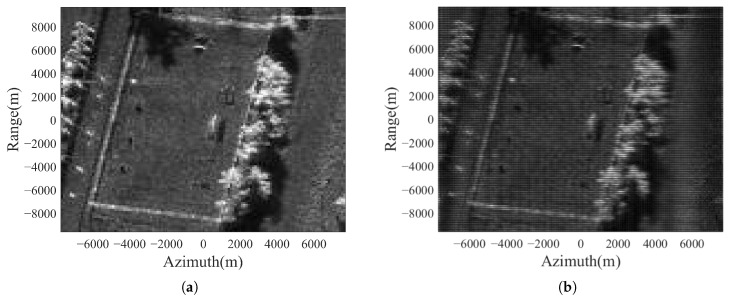
The simulation result of 2-D frequency-domain algorithm for continuous targets: (**a**) the true distribution of continuous targets, (**b**) the simulation result of continuous targets.

Then, to further evaluate the effectiveness of the proposed algorithm, we conduct a simulation using continuous targets. The simulation result is presented in Figure 13, where Figure 13a shows the true distribution of the continuous targets, and Figure 13b displays the simulated result. The structural similarity index of 0.7503 between the simulated result and the true distribution confirms that the imaging output maintains high consistency with the actual target distribution. All sub-targets within the continuous targets are well-focused. Additionally, the continuous targets exhibit a smoothing effect due to the transfer characteristics of matched filtering.

In summary, this subsection validates the effectiveness of the proposed algorithm through point target and continuous target imaging simulations.

### 4.2. Practical Implementation Feasibility Analysis

In this section, to illustrate the practical feasibility of the proposed algorithm, we evaluate it in terms of real-time processing capability and robustness against noise interference. The computational load of the proposed algorithm depends on 1-D/2-D FFT operations and phase multiplications. Let $N_{r}$ and $N_{a}$ denote the number of sampling points in the range and azimuth dimensions, respectively. As shown in Table 6, the total computational complexity of our algorithm is $O(N_{r}N_{a}log_{2}\left(N_{r}N_{a}\right))$. Since the proposed algorithm is a frequency-domain imaging algorithm, the memory consumption mainly consists of the 2-D raw data, intermediate spectra, and the final focused image. Therefore, the space complexity of the algorithm is $O(N_{r}N_{a})$. Using an i7-11800H 2.30 GHz processor for simulation, the whole processing takes 0.229147 s, and the peak memory is 4812 Kb. Overall, the proposed algorithm demonstrates near real-time processing performance.

In addition, the proposed algorithm mainly consists of FFT operations and point-wise complex multiplications. Therefore, it benefits from mature FFT IP cores implemented on FPGA, GPU or DSP processors, making real-time implementation feasible.

Additionally, to evaluate the noise robustness of the proposed algorithm, point-target simulations are conducted by adding additive white Gaussian noise to the simulated echoes at signal-to-noise ratio (SNR) levels ranging from 0 dB to 10 dB. Table 7 presents the quantitative evaluation metrics, including PSLR, ISLR, and IRW for both range and azimuth dimensions. It is observed that the imaging quality metrics exhibit stability across all tested noise levels. As the SNR increases from 0 dB to 10 dB, the IRW fluctuates from 5.2958 m to 5.3135 m, while the azimuth IRW varies from 3.4518 m to 3.4891 m. Similarly, the PSLR and ISLR for both range and azimuth directions exhibit variations smaller than 0.2 dB. In conclusion, the proposed algorithm exhibits strong robustness to noise and is therefore suitable for practical radar operating environments.

## 5. Conclusions

This paper addresses the critical challenge of imaging for the GEO SA FMCW BiSAR system with a high-maneuvering receiver, where the traditional “stop-and-go” assumption is invalid. We propose a high-precision “non-stop-and-go” echo model that accurately captures the receiver’s motion during the long propagation delay. Numerical analysis demonstrates the superior accuracy of our model over the conventional one, showing a significant reduction in both TDE and QPE. Building upon this model, a computationally efficient 2-D frequency-domain imaging algorithm is developed. By employing POSP and MSR, the algorithm effectively compensates for complex motion-induced effects, including severe range–azimuth coupling and nonlinear phase error. Comprehensive simulations, including point and distributed targets, validate the proposed algorithm. The results show that our algorithm achieves focusing quality comparable to the back-projection algorithm, while offering an improvement in computational efficiency. Meanwhile, computational complexity analysis and simulation results under different SNR levels demonstrate the feasibility of the proposed algorithm for practical implementation.

However, the current study still has several limitations. First, the present work primarily considers the vertical maneuver component; three-dimensional motion and higher-order parameters such as jerk have not been considered. Second, in the derivation of the proposed algorithm, the spatial variation effect of range has not been taken into account. Third, although additive white Gaussian noise has been evaluated, clutter, motion parameter estimation errors, and non-Gaussian disturbances remain to be investigated. Finally, although simulation results indicate that the proposed algorithm is capable of real-time processing, its performance has not been verified on embedded hardware platforms. Future work will therefore focus on extending the proposed model to three-dimensional high-maneuvering platforms, improving robustness under practical environmental and interference conditions, and implementing the algorithm on the embedded hardware processor of the radar system to verify its real-time capability.

## Figures and Tables

**Table 1:** Comparative analysis of previous studies.

Proposed	Configuration	Acceleration Included	Intra-Pulse Motion Included	Imaging Domain	Limitation
[19]	Pulsed BiSAR	Yes	No	Time	Concentration on pulsed SAR
[20]	GEO-LEO Pulsed BiSAR	Yes	No	Frequency	Non-airborne FMCW scenarios
[21]	GEO SA Pulsed BiSAR	Yes	No	Omitted	No corresponding algorithm
[26]	FMCW BiSAR	No	Yes	Frequency	Neglection of receiver motion during propagation
[27]	Spaceborne FMCW BiSAR	No	Yes	Time	High computational cost

**Table 2:** List of acronyms and descriptions.

Acronym	Description
GEO SA FMCW BiSAR	Geosynchronous Spaceborne–Airborne Frequency-Modulated Continuous-Wave Bistatic Synthetic Aperture Radar
GEO SA BiSAR	Geosynchronous Spaceborne–Airborne Bistatic Synthetic Aperture Radar
FMCW	Frequency-Modulated Continuous Wave
SAR	Synthetic Aperture Radar
GEO	Geosynchronous Earth Orbit
GEO-LEO	Geosynchronous Earth Orbit–Low Earth Orbit
LEO	Low Earth Orbit
GEO BiSAR	Geosynchronous Spaceborne Bistatic Synthetic Aperture Radar
2-D	Two-dimensional
RVP	Residual Video Phase
MSR	Method Of Series Reversion
RCMC	Range Cell Migration Correction
SRC	Secondary Range Compression
TDE	Time Delay Error
QPE	Quadratic Phase Error
POSP	Principle Of Stationary Phase
MSE	Mean Squared Error
PSLR	Peak Side-Lobe Ratio
ISLR	Integrated Side-Lobe Ratio
IRW	Impulse Response Width
SNR	Signal-to-Noise Ratio

**Table 3:** Parameters of the GEO SA FMCW BiSAR system.

Parameter Type	Parameter Name	Value	Units
FMCW Signal	Center Frequency	1.25	GHz
	Bandwidth	50	MHz
	Pulse Width	3	ms
	Pulse Repetition Frequency	150	Hz
Spaceborne Transmitter	Location	(−958, 0, 35,786)	km
	Velocity	(0,3100,0)	m/s
Receiver	Location	(0,−0.5,10)	km
	Velocity	(0,250,0)	m/s
	Acceleration	(0,0,25)	$m/s^{2}$

**Table 4:** Parameter values for algorithm accuracy and efficiency analysis of the proposed algorithm, the back-projection algorithm, and the range migration algorithm.

	Range			Azimuth			Running Time (s)
	PSLR (dB)	ISLR (dB)	IRW (m)	PSLR (dB)	ISLR (dB)	IRW (m)	
The proposed algorithm	−12.9063	−9.7356	5.3117	−12.6412	−10.2245	3.4861	0.229147
The back-projection algorithm	−12.9140	−9.6915	4.9987	−13.1310	−10.6032	2.2528	132480
The range migration algorithm	−11.4530	−9.4934	5.3192	−13.1220	−10.7580	3.5179	0.569932

**Table 5:** Evaluation parameters of point targets by the proposed algorithm.

Position	PSLR (dB)		ISLR (dB)		IRW (m)	
	Range	Azimuth	Range	Azimuth	Range	Azimuth
PT3	−12.9043	−12.6890	−9.6149	−9.0177	5.3133	3.4338
PT5	−12.9063	−12.6412	−9.7356	−10.2245	5.3117	3.4861
PT7	−12.4047	−12.7250	−9.7942	−9.0778	5.3270	3.4347

**Table 6:** The time complexity of each step in the proposed algorithm.

Step	Complexity
Dechirp	$O(N_{r}N_{a})$
RVP Compensation	$O(N_{r}N_{a})$
Azimuth FFT	$O(N_{r}N_{a}log_{2}\left(N_{a}\right))$
Residual-Phase Compensation	$O(N_{r}N_{a})$
2-D IFFT	$O(N_{r}N_{a}log_{2}\left(N_{r}N_{a}\right))$

**Table 7:** The point-target resolution parameters reconstructed via the proposed algorithm under different SNR levels.

SNR	PSLR (dB)		ISLR (dB)		IRW (m)	
	Range	Azimuth	Range	Azimuth	Range	Azimuth
0	−12.8227	−12.6059	−9.6566	−10.3342	5.3081	3.4891
5	−12.8835	−12.6220	−9.7346	−10.3659	5.3135	3.4849
10	−12.8301	−12.6156	−9.5424	−10.3088	5.2958	3.4518

## Data Availability

The original contributions presented in this study are included in the article. Further inquiries can be directed to the corresponding author.
